# Postharvest quality of two orange‐fleshed sweet potato [*Ipomoea batatas* (L) Lam] cultivars as influenced by organic soil amendment treatments

**DOI:** 10.1002/fsn3.700

**Published:** 2018-07-11

**Authors:** Richard A. Atuna, Wilberforce O. Aduguba, Abdul‐Razak Alhassan, Issah A. Abukari, Tawanda Muzhingi, Daniel Mbongo, Francis K. Amagloh

**Affiliations:** ^1^ Department of Food Science and Technology University for Development Studies Tamale Ghana; ^2^ Department of Biotechnology and Molecular Biology University for Development Studies Tamale Ghana; ^3^ Council for Scientific and Industrial Research‐Savanna Agricultural Research Institute Tamale Ghana; ^4^ International Potato Center (CIP) Food and Nutritional Evaluation Laboratory BecA Hub ILRI Nairobi Kenya

**Keywords:** cooling, evaporative, organic, poultry, storage, sweet potato

## Abstract

Two orange‐fleshed sweet potato cultivars: Apomuden and “Nane” were grown on cow dung‐, chicken manure‐, compost‐amended soils, and untreated soil. Apomuden is a variety, while “Nane” is being evaluated to be released in Ghana. The storage roots (SRs) were harvested at 3 months, cured by heaping the SRs and covering with the sweet potato foliage for 7 days in the field. The cured SRs were kept in an evaporative cool chamber to study the effect of soil amendment treatments on weight loss, rot, some nutrient composition, and sensory attributes. Boiled SRs were assessed by 70 untrained panelists after 7 weeks of storage based on the following: *general appearance, sweetness, finger‐feel firmness, and overall acceptability* using a 5‐point hedonic scale (*1 = dislike extremely* to *5 = like extremely*). Percent rot for “Nane” showed a linear trend, while that of Apomuden was nonlinear. Both cultivars showed similar trends in terms of cumulative weight loss with “Nane” recording lower weight loss compared with Apomuden. A significant (*p *< 0.001; r = 0.71) strong positive correlation was observed between weight loss and rots. “Nane” had higher dry matter (37.15% vs. 30.19%; *p *< 0.001, respectively) and starch content (59.16% vs. 51.86%; *p *< 0.001, respectively) than Apomuden. Stored SRs grown on chicken manure‐amended soil recorded the highest protein (6.41%; *p *< 0.001) and β‐carotene (16.64 mg/100 g; *p *< 0.001) content than the other treatments. There was a 35% decline in β‐carotene for Apomuden, while “Nane” increased by 24% at the end of the 7‐week storage. “Nane,” the cultivar with high dry matter content had good storage properties than Apomuden. Stored SRs cultivated on soils amended with chicken manure had higher β‐carotene and protein content. All sensory attributes ranged from 3.35 to 3.68 indicating a good consumer preference for both cultivars irrespective of the soil amendment treatment applied.

## 1 INTRODUCTION

Sweet potato (*Ipomoea batatas* (L) Lam) is an important food security crop in many developing countries including Ghana (Essilfie, Ofosu‐Anim, Dapaah, Norman, & Blay, 2015). Major production of the crop is carried out in developing countries (Crissman et al., 2007). However, over the years, there has been a decline in sweet potato yield due to the inherent poor soils in these low‐income countries (Sowley, Neindow, & Abubakari, 2015). Sweet potato is a hardy crop and can strive on marginal soils (Nedunchezhiyan & Ray, 2010). Notwithstanding its hardy nature, it still requires some important nutrients to realize its full production potential. Inorganic fertilizers may enhance good yields (Ali, Costa, Abedin, Sayed, & Basak, 2009), but farmers in low‐income countries cannot afford the costly inorganic fertilizers. Although not the focus of this study, it has been reported that the shelf life of root and tuber crops was compromised with the application of inorganic fertilizers (Biruk‐Masrie, Nigussie‐Dechassa, Abebie, Alemayehu, & Tana, 2014; Eze & Orkwo, 2010). For example, increased application of urea in carrot resulted in increased rots and visible molds (Hailu, Seyoum, & Dechassa, 2008).

Therefore, the search for cheaper soil amendments such as organic fertilizers to improve the soil fertility has become more important. Organic fertilizers improve the physical, chemical, and biological characteristics of the soil thereby increasing productivity for improved income, food, and nutrition security (Bhaskaran & Krishna, 2009; Essilfie et al., 2015; Gibberson, Joshua, Ato, Justice, & Paul, 2016). As research efforts are directed toward improving soil fertility for increased yields, it is important to consider the effect of the organic fertilizer on the storage and nutritional qualities of storage roots (SRs). For instance, soils amended with different organic fertilizers were found to have an influence on the storage qualities of two sweet potato varieties in Ghana (Essilfie, 2015). The protein content in plants depends largely on the availability of nitrogen (N) at planting, N released during the growing season, through mineralization of soil organic matter, and N applied as organic or inorganic fertilizer (Wang, Li, & Malhi, 2008).

Sweet potato generally has a short shelf life that is reported to be 7–10 days under tropical market conditions (Rees et al., 2003). This may vary depending on the cultivar and storage conditions (Ray, Ravi, Hegde, Rao, & Tomlins, 2010). However, under controlled temperature (13–15°C) and relative humidity (90%), sweet potato RSs can be stored up to one year (Woolfe, 1992). Although refrigeration is a common storage method, sweet potato and some fruits cannot be stored longer due to their susceptibility to chilling injury (Liberty, Okonkwo, & Echiegu, 2013; Olosunde, Igbeka, & Olurin, 2009). Furthermore, maintaining suitable temperature and relative humidity in tropical low‐income countries may be expensive and unsustainable (Lal Basediya, Samuel, & Beera, 2013). The current farmers’ village‐level storage methods are mostly unsatisfactory (Hayma, 2003) leading to average losses of 20–25% in sweet potato (Jenkins, 1982). In a recent study, the household‐level sandbox storage method was recommended for being able to extend the shelf life of storage roots by 2 months (Abidin et al., 2016). However, its commercial potential for improved income is limited because of the volume of SRs it can hold.

Fruits and vegetables have long been preserved using evaporative cooling system. When dry air passes over a wet surface, it causes evaporative cooling; the degree and efficiency of cooling depend on the evaporation rate and humidity of the ambient air (Liberty, Ugwuisuwu, Pukuma, & Odo, 2013). This passive cooling method differs from refrigeration or normal air conditioning because there is no external energy source. Evaporative cooling has a great potential in tropical countries like Ghana, because the harmattan season (November–March) that is characterized by warm dry air and low relative humidity (Mittal, Kataria, Das, & Chatterjee, 2006) may bring efficient evaporative cooling effect. For instance, the shelf life of garden eggs was reported to be extended by 9 days with evaporative cooling unit (Ganesan, Balasubramanian, & Bhavani, 2013).

In this study, the evaporative cooling system was adopted as a storage system to study the effect of organic soil amendment treatment on some compositional and sensory attributes of SRs.

## 2 MATERIALS AND METHODS

### 2.1 Study location and experimental design

The cultivation and curing of sweet potato were carried out in the trial fields of Council for Scientific and Industrial Research‐Savanna Agricultural Research Institute (CSIR‐SARI), Nyankpala (9° 24ˈ N, 0° 59ˈ W, 183 m above sea level), Ghana. The storage experiment was also carried out at the University for Development Studies, Nyankpala campus, Tamale (9° 25ˈ N, 0° 59ˈ W, 189 m). Both locations fall within the Guinea Savannah Agroecological Zone of Ghana.

The experimental design used was randomized complete block design arranged in a 2 × 4 factorial, thus two orange‐fleshed cultivars: Apomuden and “Nane”; four soil amendment treatments: Chicken manure, cow dung, compost, and untreated as control. The application rates for the organic manure were 10/tha as recommended elsewhere (Edison et al., 2009). The two orange‐fleshed sweet potato cultivars were Apomuden, a released variety in Ghana with a long irregular shape and a purple‐red skin color (Tumwegamire et al., 2014), while “Nane” (long oblong shape and brownish‐orange skin color), a farmer cultivar currently undergoing evaluation for release in Ghana. Both cultivars were grown to optimum maturity (3 months) before harvest.

### 2.2 Construction of zero energy cool chamber (ZECC) and storage

**Figure 1:**
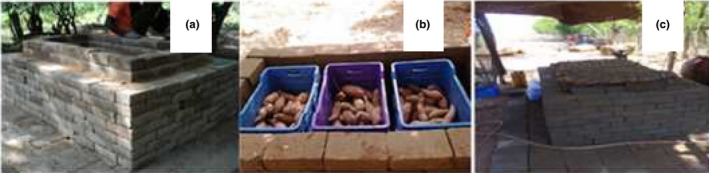
SRs in an evaporative cool chamber

A square shape platform measuring 3 × 3 m was made with cement bricks of dimension 24 × 14 × 7.5 cm. Over this platform, a double wall was erected to the height of 52 cm leaving a gap of 10 cm and wet coarse river sand was then filled in the 10‐cm gap between the walls (Figure 1a). Wire gauze and locally woven thatch cover as well as jute sack were used as top covers of the chamber. Construction of the ZECC was carried out as outlined in Roy and Khurdiya (1983).

After SRs were carefully harvested, sorted, and field‐piled cured for seven days, they were carefully transported to the storage site ensuring they were not bruised during the transportation. Further sorting was carried out to ensure only good quality SRs were stored. Fifteen kilograms (15 kg) of each cultivar from the four soil amendment treatments was weighed into plastic crates (51 × 32 × 30 cm) in triplicates. The crates filled with SRs were then randomly stored in the cool chambers (Figure 1b), ensuring each cool chamber contained six crates with two of them stacked on each other. The top covers were then used to cover the ZECC (Figure 1c).

Throughout the experimental period, the sand between the walls, bricks, and top covers of the chamber was kept moist with 100 L of water per day as recommended by Ganesan et al. (2013). The internal temperature and relative humidity of the ZECC ranged 21.1–24.9°C and 88.8–98.7%, respectively, over the storage period.

### 2.3 Weight loss, weevil damage, sprout, and rot

The weight of SRs in each crate was taken and recorded weekly. Weight loss was determined by the difference between the initial weight and final weight expressed as a percentage.

SRs were examined weekly for the presence of sweet potato weevil (*Cylas* spp.). The number of SRs damaged was divided by the total number of SRs in the crate and expressed as a percentage to obtain percent weevil damage.

The weekly number of spouted SRs was also determined and divided by the total SRs count in the crate and multiplied by 100 for percent sprouts.

SRs were assessed weekly for the incidence of rot. The number of rotten SRs was taken and recorded. The percent rot was determined as the number of rotten SRs divided by the total SR count in the crate and expressed as a percentage.

### 2.4 Compositional analysis

Freshly harvested and field‐piled cured SRs were taken for compositional analysis using the near‐infrared reflectance spectroscopy method. Samples were also taken 1 and 2 months after storage for the compositional analysis.

At least three SRs (small, medium, and big) were purposively selected for the quality analysis. The selected SRs were washed, peeled, and washed again with deionized water before quartering longitudinally and slicing it into pieces. The sliced samples about 50 g were placed into zip‐locked bags before they were put into a freezer. The samples were then freeze‐dried for 72 hr using the TK‐118 Vacuum Freeze Dryer (True Ten Industrial Company Limited Taichung, Taiwan). The freeze‐dried samples were crushed into small size and then milled into flour using a stainless steel mill (3383‐L70, Thomas Scientific, Dayton Electric Manufacturing Company Limited, Niles, IL, USA) and sieved through a 60‐mm mesh screen. The flour from the mill was collected using labeled zip‐locked bags and duly sealed until removed for analysis. About 5 g of flour of each sample was put into the cuvette and scanned for all the compositions using XDS Rapid Content Analyser (Hoganae, Sweden). The parameters analyzed include dry matter, glucose, fructose, sucrose, and starch. All determinations were assayed in triplicates.

The β‐carotene analysis was performed at the Food and Nutrition Laboratory, ILRI, Kenya. All extraction of carotenoids from OFSP fresh roots was carried out under yellow‐golden lights in FANEL. Extraction and chromatographic separation of carotenoids were performed according to the previously published methods with some modifications (Riso & Porrini, 1997). Extraction of carotenoids from OFSP fresh roots was performed using direct extraction with methanol and tetrahydrofuran (THF) as published previously (Muzhingi, Yeum, Qin, & Tang, 2008). Briefly, 1 g of OFSP fresh root grates was extracted for carotenoids by incubation with 10 ml methanol for 10 min at 85°C and vortexed for 1 min at 5 min intervals. Afterward, the mixture was homogenized for 30 s in an ice bath. The mixture was centrifuged at 800‐x g for 5 min. The methanol layer was transferred into a 50‐ml volumetric flask, and the extraction was repeated four times with 10 ml of THF, followed by vortexing and centrifugation. The THF layers were combined with the methanol layer and the volume brought up to 50 ml. One ml of the extract was dried under a gentle stream of nitrogen using an N‐Evap System (Organomation, Berlin, MA). The dried test tube contents were reconstituted in 1 ml of ethanol, sonicated for 1 min, vortexed for 30 s, and transferred into a 2‐ml HPLC vial. Then, 50 μl was injected into the HPLC system for analysis. The HPLC systems consisted of a Shimadzu CBM ‐20A Prominence Bus Module, SPD –M20A Prominence Photo Diode Array (PDA), DGU 20A5R Prominence Degasser Module, SIL 30AC Nexera Autosampler, two Nexera X2 LC 30AD pumps, a YMC Carotenoid S‐3 μm, 150 × 3.0 mm I.D column, and Shimadzu LabSolutions data management software. The HPLC mobile phase was methanol: methyl‐tert‐butyl ether: water (83:15:2, v/v/v, with 1.5% ammonium acetate in the water, solvent A) and methanol: methyl‐tert‐butyl ether: water (8:90:2, v/v/v, with 1% ammonium acetate in the water, solvent B). The gradient procedure at a flow rate of 1 ml/min was as follows: 1) 90% solvent A and 10% solvent B for 5 min; 2) a 12‐min linear gradient to 55% solvent A; 3) a 12‐min linear gradient to 95% solvent B; 4) a 5‐min hold at 95% solvent B; and 5) a 2‐min gradient back to 90% solvent A and 10% solvent B. Carotenoids were monitored at UV maximum absorption of 450 nm and DAD spectral data from 250 to 550 nm were stored to examine spectrum peaks for carotenoids. Carotenoids were quantified by determining peak areas in the HPLC chromatograms calibrated against known amounts of standards.

### 2.5 Samples preparation for sensory evaluation

Wholesome SRs about 1 kg of both Apomuden and “Nane” from the two homestead storage methods were selected into labeled net bags. The SRs were then washed and wet cooked for 20 min to become soft. The peels of the cooked SRs were removed with a knife and sliced to thumb sizes for the consumer preference test. Three figure‐coded disposable plates were used to serve the samples for scoring by the panelist. The consumer acceptability test took place at a dining room of Alimento Catering Service, University for Development Studies, Nyankpala.

### 2.6 Consumer preference test

A sensory analysis was conducted using sensory ballot. A five‐point hedonic scale, *1 = extremely dislike, 2 = dislike, 3 = neither like nor dislike, 4 = like, and 5 = like extremely,* was used to assess the sensory qualities of boiled SRs. The boiled SRs were evaluated by 70 (female = 14, male = 56) untrained panelists from the University for Development Studies. The sensory attributes evaluated were *General appearance; sweetness (sugariness); Finger‐feel firmness; and Overall acceptability*. The attribute sweetness was explained to panelist to mean desired taste as described by other researchers (Kapinga, Jeremiah, Rwiza, & Rees, 2003). Consumers rinsed their mouth with water before and in‐between samples.

### 2.7 Statistical analysis

The physical SRs quality data, rot, sprout, and weight loss, were subjected to a two‐way analysis of variance in general linear model using SAS. The rot and sprout data were square root transformed with excel (=SQRT(A2 + 0.5)) and untransformed/detransformed (=(B2*B2)‐0.5) after analysis. The weight loss data were Arcsine square root transformed (=ASIN(SQRT(D2))) and untransformed/detransformed (=(SIN(E2))*(SIN(E2))) after analysis.

A simple correlation and regression analysis were performed to establish the relationship between weight loss and rot. Moreover, a paired sample t test was used to analyze compositional quality data before and after storage in Minitab v16.2.4 (Minitab^®^ Inc. USA). The Tukey's studentized range test was used to determine which of the means was significant at (*p *< 0.05). The statistical analysis for the sensory test was performed using Microsoft^®^ Excel 2010/XLSTAT©‐Pro (version 2016.02; Addinsoft, Inc., Brooklyn, NY, USA). The Mann–Whitney test was used to analyze treatments cultivar and gender. Kruskal–Wallis nonparametric test procedure was employed to analyze the effect of the soil amendment treatment. Multiple pairwise comparisons were made using the Steel–Dwass–Critchlow–Fligner procedure/two‐tailed test when *p *< 0.05.

## 3 RESULTS

### 3.1 Physical storage root quality

**Figure 2:**
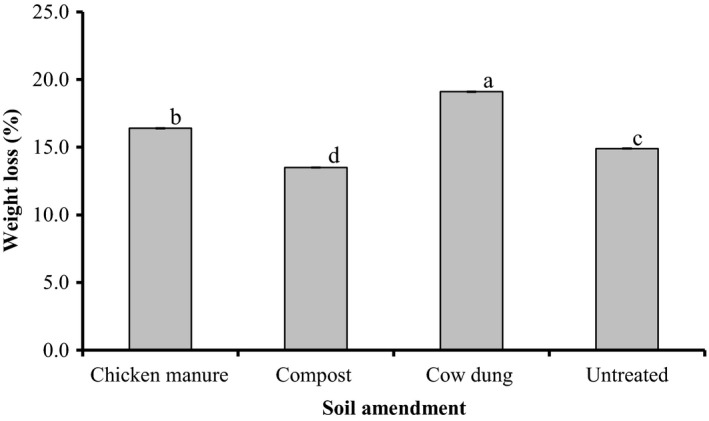
Effect of soil amendment on sweet potato SRs during a 7‐week storage period in Nyankpala, 2016. Bar values (Means ± SEM,* n *=* *3). Means with the same letters are not significantly different (*p *> 0.05)

Regardless of the cultivar type, SRs produced from the various soil amendment treatments were significantly (*p *< 0.04) different with regards to percent weight loss (Figure 2). SRs produced from compost‐amended soil recorded the least weight loss over the storage period, about 1.1, 1.2, and 1.4 times, respectively, lower than SRs grown from untreated, chicken manure‐amended, and cow dung‐amended soils.

**Figure 3:**
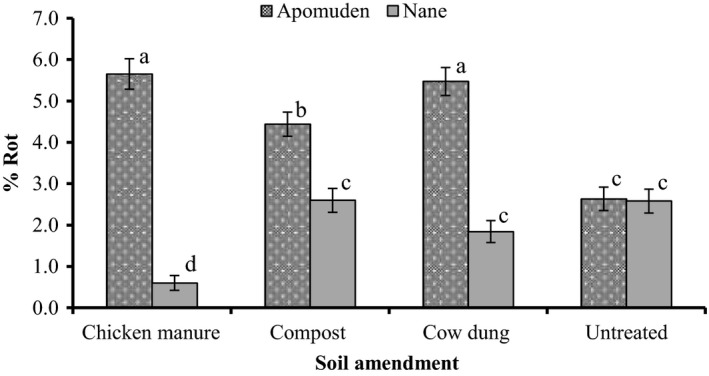
Effect of soil amendment on sweet potato SRs rot during a 7‐week storage period in Nyankpala, 2016. Bar values (Means ± SEM,* n *=* *3). Means with the same letters are not significantly different (*p *> 0.05)

Apomuden and “Nane” were significantly (*p *< 0.05) different in the various soil amendment treatment (Figure 3). For all the soil amendment treatment, percent rot was higher in Apomuden than Nane. SRs of Nane grown from chicken manure‐amended soil recorded the least percent rot compared with cow dung‐amended, compost‐amended and the no amendment.

**Figure 4:**
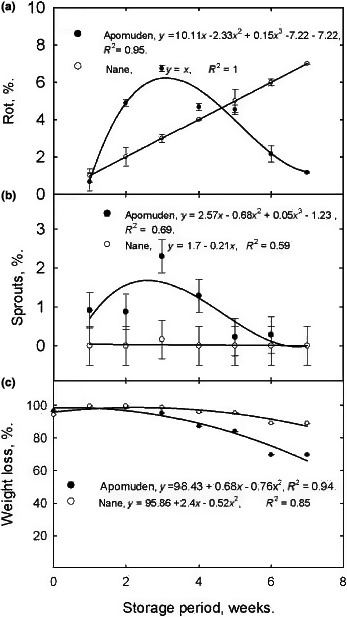
Sweet potato SRs rots (a), sprouts (b), and weight loss (c) over a 7‐week storage period in Nyankpala, 2016. Values are means ± SEM,* n *=* *3

The data (Figure 4) represent percent rot (A), sprouts (B), and weight loss (C) of SRs over 7‐week storage period in an evaporative cool chamber. Percent rot for “Nane” followed a linear trend as storage progressed (Figure 3a). About 100% of the variation in percent rot is explained by storage period. However, percent rot in Apomuden was nonlinear (cubic) with about 95% of the variation in percent rot being as a result of its association with length of storage.

Percent sprout (Figure 4b) in “Nane” was stable over the storage period. However, a unit change in storage time resulted in 0.21% decline in percent sprout with about 59% of the model being explained. On the contrary, a nonlinear (cubic) relationship was observed between percent sprout and storage period. The cumulative weight loss in both Apomuden and “Nane” followed a similar trend with “Nane” recording lower weight loss compared with Apomuden over the storage period (Figure 4c). Percent weight loss and rots were observed to have a significantly (*p *< 0.001; r = 0.71) strong positive linear relationship. This implies that, as sweet potato losses weight through transpiration and respiration, there is a high possibility of decay in those SRs.

### 3.2 Compositional quality

Soil amendment treatment significantly (*p *< 0.05) affected all SRs compositional qualities except for dry matter and starch. SRs grown from compost‐amended soil recorded the highest fructose; glucose, and sucrose content (Table 1) compared with the other soil amendment treatments. Apomuden and “Nane” differed significantly (*p *< 0.05) in all SRs compositional qualities (Table 1). The dry matter content of “Nane” (37.15%) was about 1.2 times higher than Apomuden (30.19%). As expected, the fructose, glucose, and sucrose contents were about 2.4, 2.1, and 1.2 times, respectively, lower in “Nane” compared with Apomuden (Table 1).

**Table 1:** Main effects of soil amendment treatments and cultivar on SR compositional quality on dry matter basis, Nyankpala, 2016

Treatment	Storage root compositional quality						
	Dry matter	Fructose	Glucose	Sucrose	Starch	Protein	β‐Carotene
	%						g/100 g
Chicken manure	33.60 ± 0.48^a^	2.32 ± 0.16^bc^	4.07 ± 0.20^b^	12.87 ± 0.50^b^	54.69 ± 0.59^a^	6.41 ± 0.25^a^	16.64 ± 0.50^a^
Compost	32.92 ± 0.48^a^	3.20 ± 0.16^a^	5.19 ± 0.20^a^	14.86 ± 0.50^a^	54.66 ± 0.59^a^	4.59 ± 0.25^b^	13.07 ± 0.50^b^
Cow dung	33.50 ± 0.48^a^	2.73 ± 0.16^ab^	4.65 ± 0.20^ab^	13.86 ± 0.50^ab^	55.69 ± 0.59^a^	5.34 ± 0.25^b^	11.99 ± 0.50^b^
Untreated	34.67 ± 0.56^a^	2.04 ± 0.19^c^	3.85 ± 0.23^b^	14.82 ± 0.58^ab^	56.99 ± 0.69^a^	5.04 ± 0.30^b^	12.46 ± 0.62^b^
*p*‐value	0.149	<0.001	<0.001	0.031	0.051	<0.001	<0.001
Cultivar							
Apomuden	30.19 ± 0.37^b^	3.60 ± 0.12^a^	5.98 ± 0.15^a^	15.31 ± 0.39^a^	51.86 ± 0.45^b^	5.09 ± 0.20^b^	13.90 ± 0.38^a^
“Nane”	37.15 ± 0.34^a^	1.51 ± 0.11^b^	2.91 ± 0.14^b^	12.90 ± 0.35^b^	59.16 ± 0.42^a^	5.69 ± 0.18^a^	13.18 ± 0.36^a^
*p*‐value	<0.001	<0.001	<0.001	<0.001	<0.001	<0.001	0.175

*Note*

Values represent least square means ± SEM, *n *=* *3. Least square means in the same category in a column with different letters are significantly different (*p *< 0.05).

The combined effect of cultivar and soil amendment treatment showed no significant differences (*p *> 0.05) for all compositional qualities assessed except for β‐carotene that Apomuden grown on chicken manure‐amended soil had a significantly higher (19.13 mg/100 g; *p *< 0.001) compared with the other treatment combinations (Table 2). Irrespective of the cultivar, sweet potato grown on chicken manure‐amended soils had higher β‐carotene content (14.14–19.13 mg/100 g) compared with the other soil amendment treatments.

**Table 2:** Combined effects of soil amendment treatment and cultivar on storage root compositional quality on dry matter basis, Nyankpala, in 2016

	Root compositional quality							
	%Dry matter		%Fructose		%Glucose		%Sucrose	
	Apomuden	“Nane”	Apomuden	“Nane”	Apomuden	“Nane”	Apomuden	“Nane”
Chicken manure	29.65 ± 0.68^a^	37.54 ± 0.68^a^	3.43 ± 0.23^a^	1.22 ± 0.23^a^	5.57 ± 0.28^a^	2.57 ± 0.28^a^	14.79 ± 0.71^a^	10.95 ± 0.71^a^
Compost	29.58 ± 0.68^a^	36.27 ± 0.68^a^	4.44 ± 0.23^a^	1.95 ± 0.23^a^	6.96 ± 0.28^a^	3.42 ± 0.28^a^	15.08 ± 0.71^a^	14.65 ± 0.71^a^
Cow dung	30.79 ± 0.68^a^	36.21 ± 0.68^a^	3.93 ± 0.23^a^	1.52 ± 0.23^a^	6.35 ± 0.28^a^	2.96 ± 0.28^a^	14.72 ± 0.71^a^	12.99 ± 0.71^a^
Untreated	30.76 ± 0.89^a^	38.58 ± 0.68^a^	2.72 ± 0.30^a^	1.35 ± 0.23^a^	5.03 ± 0.37^a^	2.67 ± 0.28^a^	16.63 ± 0.93^a^	13.01 ± 0.71^a^
*p*‐value	0.263		0.126		0.241		0.079	

Soil amendment treatment	Root compositional quality					
	%Starch		%Protein		β‐carotene (mg/100 g)	
	Apomuden	“Nane”	Apomuden	“Nane”	Apomuden	“Nane”
Chicken manure	50.08 ± 0.83^a^	59.31 ± 0.83^a^	6.16 ± 0.36^a^	6.67 ± 0.36^a^	19.13 ± 0.70^a^	14.14 ± 0.70^b^
Compost	51.52 ± 0.83^a^	57.80 ± 0.83^a^	4.42 ± 0.36^a^	4.77 ± 0.36^a^	13.12 ± 0.70^bc^	13.02 ± 0.70^bc^
Cow dung	52.91 ± 0.83^a^	58.48 ± 0.83^a^	4.52 ± 0.36^a^	6.17 ± 0.36^a^	10.99 ± 0.70^c^	12.98 ± 0.70^bc^
Untreated	53.99 ± 1.09^a^	61.06 ± 0.83^a^	5.26 ± 0.47^a^	4.82 ± 0.36^a^	12.34 ± 0.93^bc^	12.59 ± 0.78^bc^
*p*‐value	0.137		0.075		<0.001	

*Note*

Values represent the least square means of three samples ± SEM, *n *=* *3. Least square means in the same category in a column with different letters are significantly different (*p *< 0.05).

The data from Table 3 showed no significant (*p *> 0.05) effect of storage on the dry matter content of the SRs. However, the fructose and glucose contents of Apomuden were significantly (*p *< 0.05) influenced during storage, while the sucrose and starch contents of “Nane” were also significantly (*p *< 0.05) affected during storage. The results further revealed that the starch content of Nane declined, while there was an increase in sucrose content during storage.

**Table 3:** Changes in storage root compositional quality on dry matter basis with storage time, Nyankpala, 2016

Cultivar	Storage time	Root compositional quality					
		%Dry matter	%Fructose	%Glucose	%Sucrose	%Starch	%Protein
Apomuden	Freshly harvested	29.36 ± 0.48a	4.67 ± 0.16a	7.33 ± 0.20a	14.90 ± 0.50a	51.79 ± 0.59c	4.69 ± 0.25a
	7 Weeks After storage	31.03 ± 0.56a	2.59 ± 0.16b	4.62 ± 0.20b	15.71 ± 0.50a	51.92 ± 0.59c	5.48 ± 0.25a
“Nane”	Freshly harvested	36.99 ± 0.48a	1.64 ± 0.19c	2.82 ± 0.23c	10.17 ± 0.59b	62.17 ± 0.69a	5.39 ± 0.30a
	7 Weeks After storage	37.31 ± 0.48a	1.39 ± 0.16c	3.00 ± 0.20c	15.63 ± 0.50a	56.16 ± 0.59b	5.82 ± 0.25a
*p*‐value		0.188	<0.001	<0.001	<0.001	<0.001	0.497

*Note*

Values represent the least square means of three samples ± SEM, *n *=* *3. Least square means in the same category in a column with different letters are significantly different (*p *< 0.05).

**Figure 5:**
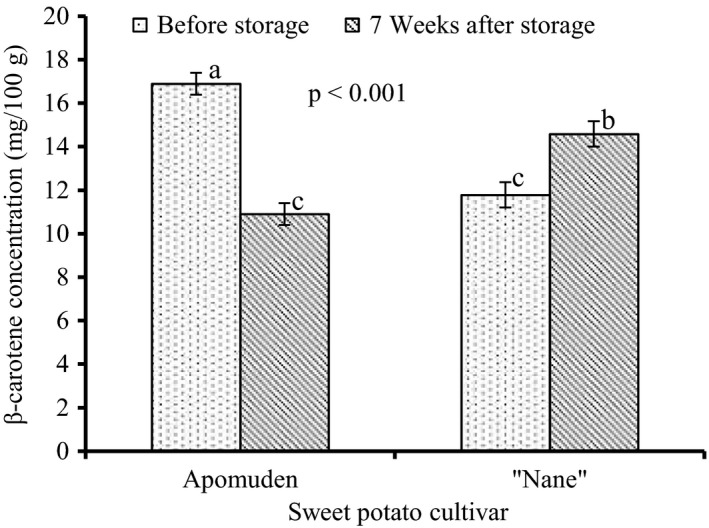
Changes in β‐carotene concentration on dry matter basis of sweet potato cultivars with storage time, Nyankpala, 2016. Bar values (least square means** **±**** SEM,* n *=* *3). Least square means with the same letters are not significantly different (*p *> 0.05)

The β‐carotene content of Apomuden and “Nane” before and after storage is presented in Figure 5. Apomuden recorded a significantly (*p *< 0.001) higher β‐carotene content relative to “Nane” before storage. However, the β‐carotene of Apomuden significantly (*p *< 0.001) declined; about 35% after storage for 7 weeks. However, the β‐carotene content in “Nane” increased by 24% after 7‐week storage.

### 3.3 Sensory quality

All sensory attributes had a sensory score above 3, an indication of good consumer preference. With the exception of general appearance, the data showed that soil amendment treatment had no significant influence (*p *> 0.05) on all sensory attributes except for general appearance that the untreated (control) had a significantly high sensory score (*p *= 0.017) compared to the other soil treatments (Table 4).

**Table 4:** Consumer acceptability test of boiled SRs after 7 weeks storage in ZECC at Nyankpala, 2016

Soil amendment*	Sensory attributes			
	General appearance	Finger‐feel firmness	Sweetness	Overall acceptability
Chicken Manure	238.53^a^	247.03^a^	256.64^a^	251.61^a^
Compost	240.76^a^	244.14^a^	234.84^a^	240.70^a^
Untreated	308.77^b^	232.47^a^	257.83^a^	249.66^a^
Cow dung	226.16^a^	251.71^a^	239.02^a^	242.17^a^
*p*‐value	<0.001	0.804	0.437	0.890
Cultivar†				
Apomuden	3.35 ± 1.04^b^	3.46 ± 1.07^a^	3.64 ± 1.07^a^	3.68 ± 0.97^a^
“Nane”	3.65 ± 1.12^a^	3.53 ± 1.10^a^	3.54 ± 1.11^a^	3.65 ± 1.08^a^
*p*‐value	0.001	0.443	0.339	0.978
Sex†				
Male (*n *=* *56)	3.51 ± 1.11^a^	3.48 ± 1.08^a^	3.59 ± 1.11^a^	3.67 ± 1.02^a^
Female (*n *=* *14)	3.59 ± 1.00^a^	3.58 ± 1.08^a^	3.53 ± 1.11^a^	3.63 ± 1.10^a^
*p*‐value	0.455	0.406	0.597	0.755

*Notes*

Means/rank means in the same column with the same letters are not significantly different (*p *> 0.05).

*Values are rank means. †Values represent the means of three samples ± SD.

Generally, there was no significant difference (*p *> 0.05) between Apomuden and “Nane” for all sensory attributes evaluated. However, “Nane” had a higher score on general appearance and finger‐feel firmness. On the other hand, Apomuden had a high sensory score in terms of sweetness and overall acceptability relative to “Nane.” Both male and females similarly (*p *> 0.05) ranked all sensory attributes assessed.

## 4 DISCUSSION

The significantly difference in weight loss of SRs grown from the various soil amendment treatment could be attributed to the difference in their nutrient composition especially that of N and K as increased preharvest application of N and K resulted in increased weight loss in potato tubers during storage (Kolbe, Müller, Olteanu, & Gorea, 1995). In another study, increased N application related directly with weight loss in the SRs of sweet potato during storage (Weston & Barth, 1997).

“Nane” recorded significantly lower rot in SRs grown from chicken manure‐amended and cow dung‐amended soil compared with untreated and compost‐amended soil. The finding agrees with the findings of Sowley et al. (2015) who reported that 80% of rot occurred in SRs grown in unfertilized soil and stored compared with poultry manure and inorganic fertilizer. However, Apomuden produced from the untreated had significantly lower rots compared with those produced from chicken manure‐amended, cow dung‐amended, and compost‐amended soils. Differences in cultivars may account for this observation.

Apomuden and “Nane” differed significantly with regards to percent rot over the storage period, and this is supported by previous studies that showed variability in decay among sweet potato cultivars (Cooley, Kushman, & Smart, 1954). The short sprouts observed during the storage of SRs in the evaporative chamber in this study could be an indication of good quality SRs as Van Oirschot et al. (2007) reported that sound or healthy SRs sprout readily. Furthermore, it is not unusual to find short sprouts (less than one‐fourth inch) during curing and storage (Chakraborty, Roychowdhury, Chakraborty, Chakravorty, & Ghosh, 2017; Edmunds et al., 2008). These short sprouts are usually broken off before SRs are sold. The results on percent weight loss are in conformity with results of earlier studies that showed that sweet potato cultivars differed widely in physiological weight loss ability (Chattopadhyay, Chakraborty, Kumar, Nanda, & Sen, 2006). For instance, cultivars with high dry matter content have low respiration rate compared with those with low dry matter content (Hirose, Data, & Quevedo, 1984; Ravi, Aked, & Balagopalan, 1996). The high moisture loss in Apomuden, the cultivar with low dry matter content, could be attributed to high respiration resulting in poor storage compared with “Nane,” the cultivar with relatively high dry matter content. The findings by Karuri and Hagenimana (1995) support the current findings as Apomuden with initially high moisture content, recorded higher degree of rot in all soil amendment treatment.

Application of organic manure generally increased the concentration of reducing sugars in sweet potato SRs. Similar findings were made in the roots of carrot by Hailu et al. (2008). However, in this study, some organic amendment resulted in higher accumulation of reducing sugar in the SRs than others. The variation in reducing sugar among the soil amendment treatment could be ascribed to differences in N source.

The high protein and β‐carotene contents of SRs grown on chicken manure‐treated soil corroborate previous works that showed that the application of N and P increased the protein and carotene content of tubers during bulking (Ezell & Wilcox, 1952; Wang et al., 2008). The high levels of N (Total *N *= 1.889%) and P (Bray I *P *= 9788.5 ppm) in the chicken manure‐amended soils could be attributed to chicken manure‐amended treatments having the highest protein and β‐carotene contents. The biosynthesis of protein and carotenoids in higher plants has been reported to be closely linked with the availability of N (Weston & Barth, 1997).

The varying β‐carotene and sucrose content between Apomuden and “Nane” could be related to cultivar difference and presents important knowledge that is relevant during processing.

Varietal difference is a major factor that determines changes in carotenoid content during storage (Ezell & Wilcox, 1952) and could be attributed to the observed findings in the current study. Thus, some sweet potato genotypes are more sensitive to storage temperature than others.

Although not significant, both fructose and glucose content of Apomuden and “Nane declined with storage and this finding lends support to (Lewthwaite, Sutton, & Triggs, 1997) who reported a decline in fructose and glucose content of SRs during storage. The finding, however, contradicts those of (Adu‐Kwarteng et al., 2014) who found a general increase in the concentration of both fructose and glucose after storage. The differences in findings could be attributed to the fact that, in the current study, maturity at the time of harvest was not taken into account unlike the previous study. The increase in “Nane sucrose during storage is in conformity with earlier findings (Adu‐Kwarteng et al., 2014; Lewthwaite et al., 1997) that showed that the sucrose content of some sweet potato cultivars generally increased with storage.

Varietal differences may account for the differences in the dry matter content as similar findings were reported elsewhere (Essilfie, 2015). The starch content of sweet potato SRs generally falls during storage, but varies among cultivars. The variation in starch content with storage is attributed to the conversion of starch to sugar (Degras, 2003). Probably, the conversion of starch into sugar was high in “Nane” resulting in rapid decline in starch content with storage compared with Apomuden.

The findings on the influence of soil amendment treatment on the sensory quality of SRs agree with Essilfie (2015) who reported that the sensory characteristics of boiled roots of two sweet potato cultivars were not remarkably affected by application of organic and inorganic fertilizer either singly or in combination. “Nane” with a higher score on general appearance and finger‐feel firmness may be due to its relatively high dry matter content compared to Apomuden. Firmness according to (2003) is an indicator of high dry matter content, a trait of sweet potato preferred by African consumers (Baafi et al., 2015; Tomlins et al., 2004). Sweetness (taste) among other attributes has been reported to be the main drivers consumer overall acceptability of a product (Kwach, Odhiambo, Dida, & Gichuki, 2010). The eating quality of sweet potato has been reported to be fundamentally linked to the sugar composition (Lewthwaite et al., 1997). Thus, the higher overall acceptability score for Apomuden may be due to its higher fructose, glucose, and sucrose content than “Nane.”

The fact that both male and female equally preferred both cultivars grown from either of the soil amendment treatment may suggest that both male and female consumers in Ghana will equally accept boiled SRs after they have been produced from either amended or unamended soils.

## 5 CONCLUSION AND RECOMMENDATION

“Nane,” the cultivar with high dry matter content, had good storage properties in evaporative cool storage than Apomuden. Stored SRs cultivated on soils amended with chicken manure had higher β‐carotene and protein content in both cultivars compared with the other treatments. Therefore, chicken manure is recommended to farmers. Storage for 7 weeks resulted in 35% decline in β‐carotene content of Apomuden and 24% increase in the β‐carotene content of “Nane.” All sensory attributes of the boiled SRs after evaporative cool storage for 7 weeks ranged from 3.35 to 3.68 indicating a good consumer preference for both cultivars irrespective of the soil amendment treatment applied.

## CONFLICT OF INTEREST

The authors declare that they have no competing interest.

## ETHICAL REVIEW

This study does not involve any human or animal testing.
